# Molecular docking and *in vitro* analysis of peptides from *Stolephorus indicus* with ACE2

**DOI:** 10.6026/97320630019531

**Published:** 2023-05-31

**Authors:** Keshapaga Usha Rani, Husain Gulam Mohammed, Surya Satyanarayana Singh, Burgula Sandeepta

**Affiliations:** 1 Department of Microbiology, Osmania University, Hyderabad - 500007; 2 National Research Institute of Unani Medicine for Skin Disorders, Ministry of AYUSH, Government of India, Hyderabad - 500038; 3 Department of Biochemistry, Osmania University, Hyderabad - 500007

**Keywords:** Peptides, cysteine protease, ACE2, hypertension, anchovies

## Abstract

Peptides from *Stolephorus indicus* (Anchovies) meat lysate were generated using a *Bacillus subtilis* cysteine protease. The peptides were generated by enzyme hydrolysis after which the hydrolysate containing peptides were analysed by LC-MS/MS. Computer aided analysis of peptides using CASTp server and GOLD software show four peptides having ACE2 inhibitory activity. Further, peptides 1 (8 amino acids), 2 (8 amino acid), 5 (9 amino acids) and 11 (12 amino acids) showed good docking features for binding to ACE2 enzyme active sites, mainly by hydrogen bonding. Peptide 1 (8 amino acids-octa-peptide) having the highest docking score was tested *in vitro* for ACE2 binding and showed up to 40 % inhibition of ACE2 activity at a concentration of 10mM. Hence, this octa-peptide has a potential role in applications involving ACE2 inhibition thereby leading to the prevention of binding of spike glycoprotein to ACE2 receptor.

## Background:

The COVID-19 pandemic caused by the Severe Acute Respiratory Syndrome Coronavirus 2 (SARS-CoV-2) virus, has triggered not only a health crisis but also a global economic disaster [1,2]. In 2003, the receptor for entry of the Severe Acute Respiratory Syndrome coronavirus (SARS-CoV) was found to be a protein called angiotensin-converting enzyme 2 (ACE2) [3]. SARS-CoV-2 also uses ACE2 receptor for its entry into the host cell. The SARS-CoV-2 surface Spike glycoprotein binds to ACE-2 and causes conformational changes in the spike glycoprotein, allowing proteolytic breakdown by the host cell transmembrane protease serine 2 (TMPRSS2), which leads to Virion internalization [4]. SARS-CoV-2 shares 79.5 % homology with SARS-CoV and 40% homology with MERS-CoV, indicating a significant genetic difference, but SARS-CoV and SARS-CoV-2 share almost 76.5% S-protein homology [5,6]. Angiotensin-converting enzyme 2 (ACE2) is a carboxy peptidase and homolog of ACE1 that is encoded by ACE2 in humans [7,8]. It is a type I trans-membrane protein composed of a cytoplasmic tail and an extracellular domain containing a HEMGH zinc-binding motif, which exhibits carboxypeptidase activity. ACE2 is expressed in vascular endothelial cells where it catalyses the conversion of angiotensin II to the vasodilatory peptide angiotensin 1-7 to regulate systemic blood pressure and angiotensin I to angiotensin 1-9, a peptide that counter-regulates the function of angiotensin II [7,9]. It is also expressed in the epithelial cells of the kidney, heart, lung, small intestine, and liver and has roles in fluid homeostasis, cardiac contractility, and amino acid absorption, as well as the prevention of pulmonary fibrosis and hypertension. ACE2 also acts as a functional receptor for severe acute respiratory syndrome coronavirus (SARS-CoV) and SARS-CoV-2 to facilitate viral entry into host cells [4,10]. Inhibitors of the ACE2 and SARS-CoV-2 interaction may be beneficial against viral infection in the treatment of COVID-19. However, such inhibitors should not affect ACE2's carboxy monopeptidase activity, as doing so could exacerbate COVID-19 comorbidities as seen in ACE2 deficient mice. Such mice exhibit increased angiotensin II-induced hypertension, susceptibility to atherosclerotic plaques, myocardial dysfunction, and insulin resistance compared with wild-type mice [11]. Therefore, ACE2 inhibition is one of the potential targets of treatment. In this study, we have screened peptides isolated from Anchovy fish hydrolysate using *Bacillus cysteine* proteases and screened them for binding to ACE2.

##  Material and Methods:

## Fish hydrolysate preparation:

Fresh Anchovy fish muscle tissue was used for preparing fish hydrolysate. Briefly, the Muscle tissue (50g) was homogenized with 200 mL of chilled 0.02M Potassium phosphate buffer, pH 8.0 and then centrifuged at 13,000 X g for 20 minutes at 4°C. The supernatant was collected immediately in sterile 50 ml tube and stored at -20°C. 

## Breakdown of meat proteins & Enzyme Hydrolysis:

50 mL of anchovy muscle tissue extract was digested with 1000 µL *Bacillus subtilis* cysteine protease enzyme using spent culture medium (Skimmed milk broth). The hydrolysate was incubated in an orbital shaker at 180 rpm at 37°C for 1 - 4 h and terminated with boiling at 100°C for 5-10 minutes respectively. The hydrolysate was cooled at room temperature for 15 min and centrifuged at 8000 X g for 30 min at 4°C. Finally, the supernatant was used as protein hydrolysate to identify the peptide sequences by Mass spectrometry (Sandor specialty diagnostics Pvt. ltd., Hyderabad, India).

## Docking:

## Domain Identification and template search:

The ACE2 sequences from *Homo sapiens* were submitted to SBASE for domain prediction [12]. The predicted domains were searched to find out the related protein structure to be used as a template by the BLAST (Basic Local Alignment Search Tool) program against PDB (Protein Data bank) [13]. The sequence that showed maximum identity with high score and e-value either zero or less negative values were aligned and was used as a reference structure. Peptides were drawn using pepdraw software and used for docking.

## Active site identification:

Active site of ACE2 from *Homo sapiens* was identified using CASTp server [14]. A new program, CASTp, for automatically locating and measuring protein pockets and cavities, is based on precise computational geometry methods, including alpha shape and discrete flow theory. CASTp identifies and measures pockets and pocket mouth openings, as well as cavities. The program specifies the atoms lining pockets, pocket openings and buried cavities; the volume and area of pockets and cavities and the area and circumference of mouth openings.

## Docking using GOLD 3.0.1:

GOLD (Genetic Optimization of Ligand Docking) a genetic algorithm (GA) based software, mainly utilizes an evolutionary strategy involving 3 genetic operators; cross overs, mutations and migrations. GOLD imports the partial flexibility to proteins and full flexibility to inhibitors. The peptides are docked into the active sites of ACE2 from *Homo sapiens* and the interaction of peptides with the active site residues are thoroughly studied using calculations of molecular mechanics. The parameters used for GA were population size (100), selection pressure (1.1), number of operations (10,000), number of island (1) and niche size. Operator parameters for crossover, mutation and migration were set to 100, 100 and 10 respectively. Default cut off values are, 3.0Å (dH-X) for hydrogen bonds and 6.0Å for van der Waals were employed. The default algorithm speed was selected and the inhibitor binding site in ACE was defined within a 10Å radius with the centroid [15]. The number of poses for peptides was set to 100 and early termination was allowed if the top three bound conformations of inhibitor was within 1.5ÅRMSD. After docking, the individual binding poses of peptides were observed and the interaction with the ACE2 was studied. The best and most energetically favourable conformation of each peptide was selected [16].

## GOLD score fitness function:

The four components viz, Protein-ligand hydrogen bond energy (external H-bond); Protein-ligand van der Waals energy (external vdw); Ligand internal van der Waals energy (internal vdw); and Ligand intramolecular hydrogen bond energy (internal- H- bond) were considered for calculating the fitness function of GOLD score. The protein-ligand hydrophobic contact was encouraged by making an empirical correction by multiplying external vdw score with 1.375. The fitness function has been optimized for the prediction of ligand binding positions.

Gold Score = S (hb_ext) + S (vdw_ext) + S (hb_int) + S (vdw_int), Where,

S (hb_ext) was the protein-ligand hydrogen bond score,

S (vdw_ext) was the protein-ligand van der Waals score,

S (hb_int) was the score from intra molecular hydrogen bond in the ligand

S (vdw_int) was the score from intra molecular strain in the ligand.

## *in vitro* ACE2 inhibitory assay:

ACE2 inhibitory activity of octapeptide1 was determined using the Cayman ACE2 inhibitory Screening Assay kit (cat No: 502100) as per the manufacturer's protocol. Briefly, 85 µL of ACE2 Assay Buffer and 5 µL of solvent was added to background wells. Whereas, 75 µL of ACE2 Assay Buffer, 10 µL ACE2 Enzyme and 5 µL of solvent was added to 100% Initial activity wells. Later, 75 µL of ACE2 Assay Buffer, 10 µL of diluted ACE2 Enzyme and 5 µL of peptide (1- 10 mM range) or the 20 µM positive control (MLN-4760) and working solution was added to inhibitor/positive control cells. To initiate the reactions 10 µL of ACE2 Substrate [Mca-APK (Dnp)] was added to all the wells being used. The mixture was mixed by gentle pipetting, covered with the 96-Well Cover Sheet followed by incubation for 30 minutes at room temperature. The reaction mixture was then read with an excitation wavelength of 320 nm and an emission wavelength of 405 nm (Tetan multimode reader, infinite M200 pro).

## Results and Discussion:

Proteases can be utilized for the generation of peptides from different food sources [17]. Such peptides showed good antioxidant, antidiabetic, antihypertensive, anticancer, and immunomodulatory properties. In Japan, fermented soya products have been recommended as a therapy for SARS-CoV-2-infected patients. In addition, several bioactive peptides from fermented Soy exhibited antiviral activity against the Influenza virus, HSV, HIV, Human respiratory illness virus and the specific antiviral peptides from Soybean revealed anti-SARS-CoV-2 therapeutic development and immunomodulatory agents *in silico* analysis [18]. Several Bacillus sp proteases have been previously used for generating industrially and medically important products such as detergents, pharmaceutics, processed leather and textiles and most importantly, biologically active peptides [19]. In the present study, a cysteine protease secreted by a *Bacillus subtilis* strain previously isolated at our laboratory was explored to generate biologically active peptides from animal meat source. We generated peptides using anchovy fish meat (*Stolephorus indicus*) as the source which has not been used previously. By the use of MALDI-TOF, the peptides generated were confirmed to be from anchovy fish as the sequences showed 100 % match with *Stolephorus indicus*. Among the list of peptides generated, 8 peptides were selected based on their larger proportions as observed in their PMF obtained after mass spectrometry.

Molecular docking is a frequently used method in computational studies to produce structure-based drugs to examine the interaction between two molecules [20,21]. For the interaction with compounds, most docking methods include rigid or flexible protein structures. In general, side chain fluctuations are taken into account in flexible docking algorithms and numerous confirmations of compounds are used to discover a better docking complex. To achieve good docking findings, a better quality protein structure is required. Even if no experimental data on structures is available, molecular docking can be used to model interactions and binding scores. In the study molecular dynamics of the docked complex were employed to improve docking conformations and correct the erroneous structural conformational shift, resulting in more accurate results.

**Figure 1:**
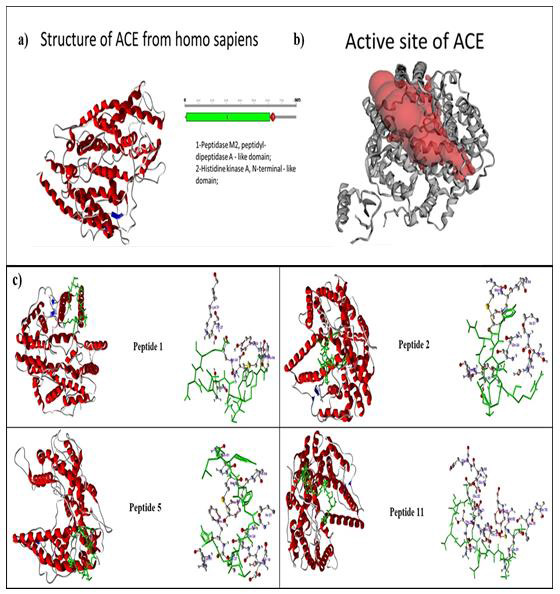
a) Collected structure of the ACE2 from *Homo sapiens* (PDB id: 1R42) from protein data bank visualized through the Discovery Studio 3.0 visualization tool. The domains of the sequence were visualized in green and red colour. The alpha helices are represented in blue colour and beta sheets in red colour. b) The big red sphere represents the cavities surrounding the active sites and was visualized using the visualization module of the Discovery studio 3.0. The binding sites were explored through the Castp server. The three putative binding sites as shown through three different coloured red balls. c) Molecular docking of peptides 1, 2, 5 and 11 with ACE2 protein using GOLD software

Docking studies were performed to gain insight into the binding conformation of pharmacophore models derived from structural manipulations onto protein. Peptides were selected based on the criteria of satisfying Lipinski's Rule-of-Five with zero violations for docking with ACE2. All docking calculations were carried out using GOLD software and the files generated were analysed for their binding conformations. Analysis was based on the Free energy of binding; Lowest docked energy and calculated RMSD values. The total clusters of docking conformations, with the docked peptides, showed positive binding energies. Among all docking conformations, the ones showing the best predicted binding free energy of peptides was selected (Figure 1). Upon docking of the peptides onto ACE2 using the Castp server, it was observed that 4 of the peptides 1, 2, 5 and 11 could bind the enzyme with a good docking scores of 25.33, 24.31, 20.2 and 23.02 respectively. The significant advantages of peptides, such as the synthesis and modification, low toxicity, high target specificity and selectivity give us an idea to design a potential therapeutic peptide candidate against ACE2. Biological properties of peptides generated from anchovy fish meat (*Stolephorus indicus*) as the protein source has not been used previously. The 4 peptides 1, 2, 5 and 11 were selected for further studies because of their strong binding to ACE2 *in silico*. These peptides show fair resistance to digestive enzymes as predicted by the peptide cutter tool on www.expasy.org and hence could be explored further. In recent times, alternative therapy for prevention of diseases such as hypertension and SARS-CoV2 infection is under extensive study. Most anti-viral and anti-hypertensive drugs have limited efficiency and have several side effects including nausea, vomiting and possible liver damage [22]. In the case of SARS-CoV2, several monoclonal antibodies such as bamlanivimab, casirivimab and imdevimab have been explored with some efficiency but these are expensive and not freely available [23]. Whereas, no single drug is useful for complete control of hypertension and diet modification is the only other way of control, which does not work on all patients. For screening for inhibition of ACE2 enzyme *in vitro*, we used octapeptide 1, since it showed the highest docking scores. We observed that octapeptide 1 showed up to 40 % inhibition of ACE2 activity. Interestingly, all 4 peptides showed anti-microbial properties upon *in silico* predictions for antibacterial, antifungal, and antiviral properties using APD3 Antimicrobial Peptide Database (Table 1, Table 2). Further screening of these peptides *in vitro* will be useful in understanding their biological activity.

## Conclusion:

Anchovy fish meat lysate was used for the generation of peptides for utilizing their biological activity. Several studies have explored animal cell lysate as a source of biologically active peptides, particularly for applications of human diseases. SARS-CoV2 has been a major pathogen in recent times, affecting millions of lives. In spite of new vaccines, anti-viral agents and other traditional medications, new treatment options are being investigated for effective treatment. Anchovy fish being a rich source of protein was explored for this purpose. The peptides generation thus were studied for their biological activity. The molecular docking scores revealed the inhibition of SARS-CoV-2 receptor binding domain i.e., ACE2 by some of the peptides. Four peptides 1, 2, 5 and 11 were predicted to bind the active site of ACE2 respectively. Among these, peptide 1, an octa peptide, showed highest docking score of 25.33. The SARS-CoV2 virus is known to enter host epithelial cells by binding to the ACE2 receptors. ACE2 inhibition via peptide binding leading to its inhibition of ACE2 receptor binding to can thus potentially prevent the virus entry into the host cell and also inhibition of ACE2 activity elevated in other pathogenic conditions such as hypertension. Interestingly all the four peptides were predicted to have antifungal or antibacterial activity as analyzed from the APD3 antimicrobial peptide database. Further studies are required for understanding the role of the peptides in modulation of ACE2 function.

## Figures and Tables

**Table 1:** Molecular docking scores of peptides with ACE2

**Ligand name**	**Docking score**	**S(hb_ext)**	**S(vdw_ext)**	**S(hb_int)**	**S(int)**	**Peptide Sequence**
pep1	25.33	38.19	38.7	0	-66.07	QWRAALDK
pep2	24.31	20.81	43.35	0	-56.11	MNGNYARR
pep3	18.75	37.76	34.16	0	-65.97	SYQPPGQR
pep5	20.2	28.6	36.12	0	-58.07	LWFGGSLGH
pep6	10.56	20.14	46.31	0	-73.26	WMIIQEMTK
pep8	12.98	20.01	39.74	0	-61.67	ELAGEPPSAR
pep11	23.02	38.37	33.67	0	-61.65	ESCDGMGDVSEK
pep12	13.57	26.35	40.62	0	-68.63	LTPYMNLTMSQK

**Table 2:** A table showing *in silico* predictions for antimicrobial properties of anchovy peptides using APD3 antimicrobial peptide database (https://aps.unmc.edu/prediction)

**S. No**	**Ligand Name**	**Peptide Sequence**	**GRAVY**	**Forms alpha helices**	**Sequence similarity (%)**	**Matched AM Peptide**	***k*Predicted AMA**
1	Peptide 1	QWRAALDK	-1.11	Yes	36.36	AP02411 (Balteatide)	Antibacterial, antifungal
2	Peptide 2	MNGNYARR	-1.75	Yes	44.44	AP01226 (microcin C7)	Antibacterial
3	Peptide 5	LWFGGSLGH	0.47	No	46.15	AP02583 (Temporin-1S)	Antifungal
4	Peptide 11	ESCDGMGDVSEK	-0.975	Yes	41.6	Delphtibactin-A (AP03142)	Antibacterial
